# Right Thoracotomy Minimally Invasive AVR: Use of Preoperative CT Scan to Plan Incision

**DOI:** 10.1186/1749-8090-10-S1-A28

**Published:** 2015-12-16

**Authors:** Arthur Martella, Steven Kernis, Steven Curiale, Uche Ndubizu, Jane Cichelli

**Affiliations:** 1 Our Lady of Lourdes Medical Center, 1600 Haddon Ave., Camden, NJ 08103, USA

## Background/Introduction

Aortic valve surgery through a right anterior mini-thoracotomy (MT) has demonstrated excellent short-term and long-term-term results, becoming a feasible alternative to the sternotomy approach. Widespread use is limited by unpredictable exposure of the aortic valve. Current use of CT measurements of the aorta to the sternum is not reliably predictive of exposure and frequently excludes patients from a minimally invasive approach.

## Aims/Objectives

Use of TAVR CT scan imaging to identify the both the position and orientation of the aortic valve should predict the location of the incision.

## Method

All isolated aortic valve procedures are approached through a MT. From January 2013 to October 2014, 100 consecutive miniAVR patients were evaluated by TAVR CT scan imaging and the orientation of the aortic valve annulus was determined. Based on these results, the right thoracotomy was positioned medial, midclavicular or lateral. Intraoperative and 30 day results were analyzed and compared to the 50 prior mini-AVRs. All patients received HTK (histadine-tryptophan-ketoglutarate) cardioplegia.

## Results

Median patient age was 68 years (range: 44-89), 21 patients were 80 years or older, 3 patients had reoperative aortic valve surgery and 14 had percutaneous coronary intervention prior to surgery (hybrid procedure). Cross clamp time (102 v. 118 min.), cardiopulmonary bypass time (126 v. 148 min.) and implant time (78 v. 101 min.) were all reduced in the study group compared to the historical control group. Length of stay was similar in both groups (5.1 v. 5.3 days). There was one return for bleeding in each group and one 30 day mortality in the control group.

## Discussion/Conclusion

Minimal access approaches in aortic valve surgery are safe and feasible with excellent outcomes. From our TAVR experience, we have learned that the aortic root anatomy varies significantly. Placement of the incision perpendicular to plane of the aortic valve allows for more predictable visualization of the valve and may allow the procedure to be offered to higher risk and obese patients. Patients with very anterior and medially placed aortas may be the most challenging group.

